# 2,5-hexanedione downregulates nerve growth factor and induces neuron apoptosis in the spinal cord of rats via inhibition of the PI3K/Akt signaling pathway

**DOI:** 10.1371/journal.pone.0179388

**Published:** 2017-06-27

**Authors:** Zhemin Wang, Zewen Qiu, Chenxue Gao, Yijie Sun, Wei Dong, Yan Zhang, Ruolin Chen, Yuan Qi, Shuangyue Li, Yanjie Guo, Yongjun Piao, Sheng Li, Fengyuan Piao

**Affiliations:** 1 Department of Occupational and Environmental Health, Dalian Medical University, Dalian, Liaoning, China; 2 Laboratory Animal center, Dalian Medical University, Dalian, Liaoning, China; 3 Department of Sexually Transmitted Disease, Heping Center for Disease Control and Prevention of Tianjin, Tianjin, China; 4 Department of Biochemistry, Dalian Medical University, Dalian, Liaoning, China; 5 Department of Dermatology, the First Affiliated Hospital of Dalian Medical University, Dalian, Liaoning, China; Institute of Biochemistry and Biotechnology, TAIWAN

## Abstract

2,5-hexanedione (2,5-HD) is the main active metabolite of n-hexane and induces apoptosis in nerve tissue, however, the mechanism of which remains unclear. In the present study, neuropathic animal models were successfully constructed in rats by injecting 100, 200 and 400 mg/kg 2,5-HD intraperitoneally for 5 weeks. Rats exposed to 2,5-HD exhibited progressive gait abnormalities and slower motor neural response in a dose-dependent manner. TUNEL analysis and immunofluorescence dual labeling revealed that the spinal cord of the 2,5-HD treated rats underwent significantly more apoptosis in the cells of spinal cord than that of the control group. The neuron apoptosis index in spinal cord was 4.1%, 6.7%, 9.8% respectively in rats exposed to 100, 200 and 400 mg/kg 2,5-HD, compared with 1.1% in the control group (*p < 0*.*05*). Biochemical analysis showed that 2,5-HD exposure downregulated NGF expression in the spinal cord of the intoxicated rats; inhibited the phosphorylation of Akt and Bad, two key players in PI3K/Akt pathway downstream of NGF; increased the dimerization of Bad with Bcl-xL in the mitochondrial fraction, followed by the release of cytochrome c and activation of caspase-3 in the spinal cord of rats. In vitro study showed that the NGF expression decreased significantly in VSC4.1 cells dosed with 5.0, 10.0 mM 2,5-HD in comparison with the control group. It was also found that NGF supplement repressed the induced apoptosis, and increased p-Akt and p-Bad level in 2,5-HD treated VSC4.1 cells, which could be antagonized by PI3K kinase (the upstream member of Akt) inhibitor LY294002. Taken together, our experimental results indicate that 2,5-HD may induce apoptosis in the spinal cord of rats via downregulating NGF expression and subsequently repressing PI3K/Akt signaling pathway.

## Introduction

In recent years, the health threat of n-hexane, a widely used organic solvent in various industries, is receiving more and more attention. Occupational toxicological researches showed that n-Hexane causes serious harm in workers, and the nervous system is the major target of n-hexane [1–2]. It has been documented that occupational or experimental chronic exposure to n-hexane produces noticeable nerve tissue damage [3–5]. Occupational contact with n-hexane resulted in numbness and tingling sensation in the toes and fingers, followed by progressive weakness and areflexia, particularly in the distal limbs [6]. Severely intoxicated cases may result in drop foot, claw hand and spastic gait [7]. Electrophysiological studies demonstrate marked slowing of motor nerve conduction velocity (MCV), focal conduction block with temporal dispersion of compound muscle action potentials and marked prolongation of distal latencies, particularly in the lower extremities in intoxicated patients [8–10]. The causative agent of n-hexane has been determined as its metabolite 2,5-hexanedione (2,5-HD) [11]. In vitro studies showed that 2,5-HD induced nervous cell apoptosis. Ogawa et al. [12] reported that having been treated with a low concentration of 2,5-HD, dorsal root ganglion cells of murine origin underwent cell apoptosis. In rats exposed to 2,5-HD the total number and the size of neurons were markedly reduced [13]. Zilz et al. [14] reported that 2,5-HD induced apoptosis in human neuroblastoma line SK-N-SH. These results suggested that apoptosis induced by 2,5-HD in nervous system may account for the neurotoxicity. However, the mechanism underlying 2,5-HD-induced neuronal apoptosis is still elusive and the scenario in vivo requires investigation.

Neurotrophins are important for neural survival, which are secreted by nerve tissue and prevent the associated neuron from initiating apoptosis [15]. Nerve growth factor (NGF) is the most abundant neurotrophin in central nervous system (CNS) [16]. Deficit in the production and utilization of NGF leads to a variety of CNS dysfunctions [17]. Liu et al. [18] examined the expression level of NGF in VSC4.1 motor neuron cells exposed to 2,5-HD, and found that the endogenous NGF level was remarkably decreased in the experimental group, indicating that NGF may be a toxic-interfered target of some neurotoxicants including 2,5-HD.

NGF has been found to activate Akt and to promote cell survival through the PI3K/Akt pathway. NGF administration prevented thapsigargin-induced apoptosis in PC12 cells via activation of the PI3K/Akt signaling pathway [19]. NGF withdrawal causes a rapid decrease in PI3K and Akt activity, which activates the mitochondrial pathway of apoptosis in sympathetic neurons cultured in vitro [20]. These results indicated that abnormal changes of NGF level in nerve system might have adverse effect on activation of PI3K/Akt signaling pathway. Akt is a main effector in the PI3K/Akt signaling pathway. Phosphorylation of Akt inhibits apoptosis and inhibition of the Akt phosphorylation induces apoptosis [21]. After phosphorylation, Akt functions through phosphorylation and inhibition of Bcl-2-associated death promoter (Bad) [22–23]. Phosphorylated Bad then binds cytosolic protein 14-3-3 to release anti-apoptotic protein Bcl-xL, which together with Bcl-2 can block pro-apoptotic protein Bax translocation to the mitochondria, maintain mitochondrial membrane potential (MMP), and prevent the release of cytochrome c from the mitochondria and the subsequent apoptosis [24–27]. Therefore, it is interesting to ask whether 2,5-HD induces apoptosis via PI3K/Akt signaling pathway, whether 2,5-HD represses NGF expression, and whether the repressed NGF expression may be involved in 2,5-HD-induced apoptosis via inhibiting PI3K/Akt signaling pathway.

In the present study, using a 2,5-HD-intoxicated rat model, we found that 2,5-HD exposure induced cell apoptosis in spinal cord, which was accomplished by inhibiting phosphorylation of Akt and Bad. Moreover, it was found that dimerization of Bad with Bcl-xL was increased in the mitochondrial fraction, followed by the release of cytochrome c and activation of caspase-3 in spinal cord. NGF level was found to be reduced at both mRNA and protein levels after 2,5-HD exposure. NGF supplement repressed the induced apoptosis, and increased p-Akt and p-Bad level in 2,5-HD treated VSC4.1 cells. This protective effect of NGF was antagonized by PI3K kinase (the upstream member of Akt) inhibitor LY294002. These results indicate that 2,5-HD induces apoptosis in nerve tissue via PI3K/Akt signaling pathway and represses NGF expression. It is also suggested that the PI3K/Akt-involved apoptosis maybe associated with NGF repression.

## Materials and methods

### Chemicals and antibodies

2,5-HD (purity > 99%) and Hoechst 33342 were purchased from Sigma-Aldrich Co. LLC. (St. Louis, Missouri, USA). Rabbit polyclonal anti-NGF, anti-MAP2, anti-Akt, anti-p-Akt (ser 473), anti-p-Bad (ser 136) and mouse polyclonal anti-Bad were purchased from Cell Signaling Technology, Inc. (Danvers, MA, USA). Rabbit polyclonal anti-Bcl-xL, mouse polyclonal anti-cytochrome c and VDAC were purchased from Abcam Inc. (Cambridge, MA, USA). Goat polyclonal anti-Choline O-acetyltransferase (ChAT) was purchased from Millipore, (Bedford, MA, USA). Mouse monoclonal anti-NGF was purchased from Santa Cruz Biotechnology (Santa Cruz, CA, USA). Alexa-Fluor 594 conjugated donkey anti-rabbit IgG, Alexa-Fluor 594 conjugated donkey anti-goat IgG, Alexa-Fluor 488 conjugated goat anti-mouse IgG and Alexa-Fluor 488 conjugated donkey anti-mouse IgG were purchased from Jackson ImmunoResearch (West Grove, PA, USA). Mouse polyclonal anti-β-actin, goat-anti-rabbit horseradish peroxidase (HRP)-conjugated IgG and goat-anti-mouse horseradish peroxidase (HRP)-conjugated IgG were purchased from ZSGB Biotechnology, Inc. (Beijing, China). RIPA lysis buffer, BCA Protein assay Kit, ECL enhanced chemiluminescence kit were purchased from Beyotime Biotechnology, Inc. (Shanghai, China). All other chemicals were of the highest grade commercially available.

### Ethics statement

This study was performed in accordance with the Animal Guideline of Dalian Medical University and in agreement with the Ethical Committee of Dalian Medical University (Permit Number: SCXK 2008–0002). All efforts were made to minimize the number of animals used and to ameliorate their suffering. Adequate and standard measures were taken to minimize pain and discomfort taking into account human endpoints for animal suffering and distress.

### Animal treatment

Forty adult male SD rats, weighing 200∼230g, were purchased from the Experimental Animal Center of Dalian Medical University. The rats were housed in polycarbonate boxes, with sufficient drinking water and food. The animal room was maintained at approximately 22°C and 50% relative humidity with a 12 h light-dark cycle. All surgeries of perfusion were performed under anesthesia to minimize pain and distress in the experimental animals. They were housed in the Experimental Animal Center of Dalian Medical University for 7 days for acclimatization, and randomly divided into four groups (n = 10 per group). Experimental group rats were treated with 2,5-HD by intraperitoneal injection (i.p.) at a dosage of 100, 200 and 400 mg/kg/day (five times per week), according to Torres et al. and Cui et al [28–29]. 2,5-HD was dissolved in 0.9% saline and administered at 3 ml/kg body weight/dose. The corresponding control group rats received an equivalent volume of 0.9% saline by i.p. The health conditions of the rats were monitored twice per day throughout the experimental procedure. We had two scoring criteria to determine whether to euthanize the rats: intake food score and gait score.

The rats in the experiment were housed in separate cages. The weights of food were measured every 24 hours (at 9 am each day) and the difference of the data was calculated as the food weight consumed by the rat that day. Food intake score was assigned from 1 to 4. 1: normally intake food (81%-100% of the original food intake. Food intake per day by the rat before intoxication was set as 100% and named as original food intake); 2: impaired intake food (41%-80% of the original food intake); 3: barely intake food (10%-40% of the original food intake); 4: unable to intake food (less than 10% of the original food intake).

Gait socre: 1: a normal, unaffected gait; 2: a slightly affected gait (tip-toe walking, slight ataxia, and hindlimb weakness); 3: a moderately affected gait (obvious movement abnormalities characterized by dropped hocks and tail dragging); 4: a severely affected gait (frank hindlimb weakness and inability to rear).

If the total score (intake food score + gait socre) reached 8, we euthanized the rat by administering 10% chloral hydrate (3.5 ml/kg, i.p.) for anesthesia, and then putting the rat in a CO_2_ filled glass container. During the experimental procedure, one rat was euthanized prior to the experimental endpoint in the 400 mg/kg group, because the total score of the rat reached 8. No other unexpected mortality was observed. In order to render the poisoning rats as comfortable as we can afford for humane reason, we handled all rats gently; especially took the seriously ill rats out in individual cages, and observed them more frequently.

The onset and development of neurotoxicity were determined by neurological testing once per week, the changes of motor nerve conduction velocity were also measured at the same time. On completion of 5 weeks of 2,5-HD administration, most rats of each group appeared their symptoms in different degrees, animals were killed and spinal cord was rapidly extracted. The persons who perform subsequent biochemical analysis were intentionally kept blind to the grouping of the rats.

### VSC4.1 motoneuron cell culture

Ventral spinal cord 4.1 (VSC4.1) motor neurons were constructed by fusing embryonic rat ventral spinal cord neuron with mouse N18TG2 neuroblastoma cell [30–31]. VSC4.1 motoneurons were grown in monolayer to subconfluence in poly-L-ornithine coated 75 cm^2^ flasks containing 10 ml of DMEM medium with 15 mM HEPES, pyridoxine, and NaHCO_3_ (Sigma, St. Louis, Missouri, USA), supplemented with 2% Sato’s components, 1% penicillin and streptomycin (Beyotime, Shanghai, China), and 15% heat-inactivated fetal bovine serum (Hyclone, Logan, UT, USA). Cells were grown in an incubator at 37°C with 5% CO_2_ and full humidity.

### Neurological testing

2,5-HD-induced neurological defects were detected and quantified using gait score, which was chosen because it represents sensitive and reliable indices of toxicant-induced changes in neurological status. To measure gait abnormalities, the rats were placed in a clear plexiglass box and were observed for 3 min [4,32]. Following observation, a gait score was assigned from 1 to 4, where 1: a normal, unaffected gait; 2: a slightly affected gait; 3: a moderately affected gait; and 4: a severely affected gait. A trained, test-blinded observer who was not involved in animal care or 2,5-HD exposure performed the behavioral evaluation. Three successive measurements were averaged for each 2,5-HD-intoxicated or control rats [33].

### Motor nerve conduction velocity

The conduction velocity of the peripheral nerve was measured in the rat's tail using Electromyogram Evoked Potential Instrument NTS-2000 (Nuocheng Electric Co. Ltd by Shares, Shanghai, China). The rat was wrapped in a towel to keep it immobilized without anaesthesia and laid on its back to allow electrodes to be inserted in the tail at the points.

Electrode A was inserted 2 cm distal from the anus, electrode B, 7cm proximal to A, and electrode C, 5 cm proximal to B. The references were inserted about 2 mm from each electrode, and the body earth was inserted between A and B. A was the stimulation point, B and C were the record point. The electrodes were stainless steel needles, 0.34mm in diameter and about 15 mm long. After the insertion of the electrodes, the tail was immersed in a paraffin bath with the temperature maintained between 37 and 38°C. In the experiments of motor nerve conduction velocity, well-trained technicians were in charge of measurement to make the measurement time as short as possible and to minimize suffering and distress of the rats. The conduction velocity of the tail nerve was measured more than four minutes after immersion, and the measurement was finished within 20 minutes, as in the previous experiment. The tail nerve was stimulated by a square pulse of 0.3 msec duration, 1 Hz and the supramaximal electric current with an electro stimulator is 10 mA. The biopotentials were observed [34].

For each measurement, the rat was stimulated 3 times at point A and recorded at point B per animal, and stimulated another 3 times at point A and recorded at point C. Mean values were calculated and used in the formulae:

1. Motor nerve conduction velocity (MCV) = distance (BC)/latency time (AC-AB);
2. Distal latency (DL) = latency time (AB).

### TUNEL assay

For analysis of cell apoptosis in the rat spinal cord, the rats were anesthetized with 10% chloral hydrate (3.5 ml/kg, i.p.) and perfused with 0.9% NaCl, followed by 4% paraformaldehyde in 0.01M PBS (PH = 7.4). Spinal cord was removed, post-fixed in cold 4% paraformaldehyde overnight, and embedded in paraffin, cut into 5 μm slices. The slides of lumbar vertebra were used for the subsequent staining experiments. Then slides were rinsed sequentially in 100%, 95% and 75% ethanol, rehydrated, put into 10 mmol/L citrate pH 6 in at 95°C water bath for 30 minutes for permeabilization and further digested with 1 μg/ml proteinase K for 10 minutes at 37°C. TUNEL was performed using in Roche’s In Situ Cell Death Detection Kit, Fluorescein (Roche, Germany). After rinsing in PBS twice for 10 min, the area around the sample was dried, and the sections were incubated with the TUNEL reaction mixture for 60 min at 37°C in a humidified atmosphere in the dark. Hoechst 33342 (2 mg/ml in PBS) was used to counter stain the nuclei. The slides were rinsed three times in PBS to stop the reaction. Apoptosis-positive cells were counted in three slices per rat.

To investigate the colocalization of the neural marker MAP2 with TUNEL, double staining of MAP2 with TUNEL was performed, the sections were blocked in 5% normal donkey serum (Jackson ImmunoResearch) and incubated with polyclonal rabbit anti-MAP2 antibody (1:500; Cell Signaling Technology) at 4°C overnight, then reacted with Alexa-Fluor 594 conjugated donkey anti-rabbit (1:600; Jackson ImmunoResearch) at room temperature for 1 h. Then the sections were examined TUNEL positive cells performed using in Roche’s In Situ Cell Death Detection Kit and use Hoechst 33342 (2 mg/ml in PBS) to counter stain the nuclei. MAP2^+^/TUNEL^+^ cells were counted in three slices per rat.

For analysis of cell apoptosis in VSC4.1, cells cultured on 3.5 cm cell culture dishes were washed with PBS, fixed in 4% paraformaldehyde, and treated with permeabilization solution (0.1% Triton X-100 in 0.1% sodium citrate) for 2 min on ice. Afterward, the samples were then incubated with the TUNEL reaction mixture for 60 min at 37°C in a humidified atmosphere in the dark, then stained with Hoechst 33342 (2 mg/ml in PBS) for 5 min. The cells were rinsed 10 min × 3 in PBS to stop the reaction.

For experiments above, cells were observed using a fluorescence microscope (×400 magnification). Six fields were randomly selected and the percentage of positive cells was calculated as the apoptosis index (AI) using the following equation: AI = (number of positive cells / total number of cells) × 100%.

### Immunofluorescence dual staining

To investigate the colocalization of the motor neuron marker ChAT with NGF, double staining of ChAT with NGF was performed, the sections were blocked in 5% normal donkey serum (Jackson ImmunoResearch) and incubated with polyclonal goat anti-ChAT antibody (1:150; Millipore, Bedford, MA, USA) at 4°C overnight, then reacted with Alexa-Fluor 594 conjugated donkey anti-goat (1:200; Jackson ImmunoResearch) at room temperature for 1 h. The sections were further incubated with monoclonal mouse anti-NGF antibody (1:200; Santa Cruz) at 4°C overnight, followed by incubation with Alexa-Fluor 488 conjugated donkey anti-mouse (1:200; Jackson ImmunoResearch). Finally, the sections were observed under a fluorescence microscope.

### Quantitative real-time PCR analysis

Total RNA was extracted from rat spinal cord of lumbar vertebra by using RNAiso Plus (Takara, Tokyo, Japan) according to the manufacturer’s instructions. The RNA was quantified by using a spectrophotometer. Only RNA samples with an A260/A280 between 1.8 and 2.2 were used for reverse transcription. One microgram of total RNA was reverse transcribed using a Reverse Transcription Kit (Takara, Tokyo, Japan) in T100™ Thermal Cycler (Bio-rad, Hercules, USA). The reverse conditions were as follows: 37°C for 15 min, 85°C for 5 s, and then the reverse-transcription was staged in 4°C. Real-time Q-PCR was performed with a SYBR Green PCR kit (Takara, Tokyo, Japan) using the TP800 Real-Time PCR Detection System (Takara, Tokyo, Japan). The primers for NGF and β-actin are shown in Table 1 (designed by Takara, Dalian, China). The reaction conditions were as follows: an initial denaturation at 95°C for 5 min, followed by 40 cycles of 95°C for 30 s, 55°C for 30 s, and 72°C for 30 s. The comparative cycle threshold (Ct) method was used here to calculate the expression of genes with reference to β-actin mRNA as an internal control. The relative mRNA levels of target genes were presented as a ratio to control values.

**Table 1:** Specific primer sequences used in real-time RT-PCR.

Gene	Primer	Sequences
**NGF**	Forward	5’- TGCCCCTGCTGAACCAA-3’
	Reverse	5’-GCTTGCTCCTGTGAGTCCTGT -3’
**β-actin**	Forward	5’-GGAGATTACTGCCCTGGCTCCTA-3’
	Reverse	5’-GACTCATCGTACTCCTGCTTGCTG-3’

### Western blot analysis of Akt, Bad, p-Akt, p-Bad and NGF expression

For Analysis of the proteins, the rat spinal cord of lumbar vertebra was homogenized in ice-cold RIPA Tissue Protein Extraction Reagent (Beyotime, Shanghai, China) supplemented with 1% proteinase inhibitor mix and incubated at 4°C for 1 h. After incubation, debris was removed by centrifugation at 12,000 g for 15 min at 4°C, and the lysates were stored at -80°C until used. The total protein concentration in the lysates was determined using a BCA protein assay kit (Beyotime, Shanghai, China).

For analysis of proteins in VSC4.1, cells were homogenized in ice-cold RIPA Tissue Protein Extraction Reagent (Beyotime, Shanghai, China) supplemented with 1% proteinase inhibitor mix and incubated at 4°C for 1 h. After incubation, debris was removed by centrifugation at 14,000 × g for 15 min at 4°C and the lysates were stored at -80°C until used. The total protein concentration in the lysates was determined using the BCA protein assay kit (Beyotime, Shanghai, China).

The proteins (100 μg per lane) were mixed with an equal volume of SDS-PAGE loading buffer, separated by SDS-PAGE under no-reducing conditions using 12% SDS-PAGE gels, and then electrotransferred to PVDF membranes (Millipore, Temecula, USA). The membrane was blocked by 5% skimmed milk in TBST for 1 h and then incubated overnight at 4°C with rabbit anti-rat NGF (1:1000), Akt (1:500), Bad (1:500), p-Akt (ser473) (1:1000), p-Bad (ser136) (1:1000) and mouse anti-rat β-actin (1:500). The membrane was washed three times with TBST for 15 min and then incubated at room temperature for 1 h with horseradish peroxidase-conjugated goat anti-rabbit IgG (1:5000) or horse-radish peroxidase-conjugated goat anti-mouse IgG (1:5000). The signals were visualized using an ECL enhanced chemiluminescence kit and quantified densitometrically using ChemiDoc™ XRS+ System (Bio-rad, Richmond, USA). All proteins were expressed as the ratio of the β-actin protein.

### ELISA assay of NGF content

The amount of NGF in spinal cord of lumbar vertebra was determined using an indirect sandwich enzyme-linked immunosorbent assay (Millipore, Temecula, USA) as previously described [35–36]. Briefly, 96-well ELISA plates were coated with a monoclonal anti-NGF antibody diluted in carbonate coating buffer (pH 9.7) and incubated overnight at 4°C. Plates were then blocked using 1 × blocking buffer (200 μl/well) for 1 h at 20°C. Following incubation, NGF standards (0–1000 pg/well) or diluted medium (100 μl) were added to plates and incubated for 2 h at 20°C. After the washes, the plates were incubated with a monoclonal rat anti-NGF antibody overnight at 4°C. After a second round of washes, the plate was incubated with horseradish peroxidase-conjugated anti-rat antibody (1:1000) for 2 h at 20°C. The plates were again washed and incubated with enzyme substrate (TMB/E, Millipore, Temecula, USA) for 10 min at 20°C. The enzyme reaction was stopped by adding stop solution (100 μl/well) and the absorbance was measured at 450 nm by a microplate reader (Multiskan Ascent, Thermo, Waltham, USA). The detection limit was 10 pg/ml.

### Isolation of mitochondrial and cytosol protein

Mitochondria were isolated from spinal cord of lumbar vertebra using Tissue Mitochondria Isolation Kit (Beyotime, Shanghai China) according to the manufacturer’s protocol. Spinal cord of lumbar vertebra was homogenized in Mitochondria Isolation Reagent A at a ratio of 10 μl/mg tissue. Homogenized samples were centrifuged at 600 g for 5 min at 4°C, and the supernatant obtained was subsequently centrifuged at 11,000 g for 10 min. The pellet was suspended, the supernatant (cytosol fraction) was transferred to a new tube.

### Coimmunoprecipitation

Rat spinal cord of lumbar vertebra was collected and mitochondrial fraction was extracted as described above. Approximately 100 μg from the mitochondrial fraction were used for coimmunoprecipitation. The protein sample was mixed with 20 μl protein A+G sepharose (Beyotime, Shanghai, China) and incubated for 30 min at 4°C. Then the samples were centrifuged at 12,000 g for 10 min. The supernatant was incubated with 2 μg polyclonal rabbit anti-Bcl-xL antibody (1:1000) and 15 μl of protein A+G sepharose (50% slurry) for 5 h at 4°C. After centrifugation at 12,000 g for 1 min, the supernatant was subjected to Western blot analysis with anti-Bad antibody (1:500).

### Determination of cytochrome c protein expression

To obtain cytosolic and mitochondrial fractions, spinal cord of lumbar vertebra was collected, cytoplasmic and mitochondrial fractions were extracted with a tissue mitochondria isolation kit (Beyotime, Shanghai, China) as mentioned above. The proteins (20 μg/lane) were mixed with an equal volume of SDS-PAGE loading buffer and separated by SDS-PAGE under non-reducing conditions using 10% SDS-PAGE Gels, and then electrotransferred to Hybond-P PVDF membrane. The membrane was blocked with blocking buffer containing defatted milk power for 1 h and incubated overnight at 4°C with mouse anti-rat cytochrome c polyclonal antibody (1:100). Rabbit anti-rat VDAC (1:1000) and mouse anti-rat β-actin (1:500) were used as loading control for mitochondrial proteins and cytosolic proteins respectively. The membrane was washed three times with Tris buffered saline containing 0.05% Tween-20 (TBST) for 10 min and then incubated at room temperature for 1 h with horseradish peroxidase-conjugated goat anti-mouse IgG (1:5000). The signals were visualized using an ECL enhanced chemiluminescence kit (Beyotime, Shanghai, China) and quantified densitometrically using UVP BioSpectrum Multispectral Imaging System (Ultra-Violet Products Ltd. Upland, CA, USA).

### Determination of caspase-3 activity

The activity of caspase-3 was determined using the Caspase-3 activity kit (Beyotime, Shanghai China) according to the manufacturer’s protocol. Briefly, protein was isolated from spinal cord using the lysis buffer supplied with the kit. Assays were performed on 96-well microtitre plates by incubating 40 μl protein of lysate per sample in 50 μl reaction buffer (1% NP-40, 20 mM Tris-HCl (pH 7.5), 137 mM NaCl and 10% glycerol) containing 10 μl caspase-3 substrate (Ac-DEVD-pNA) (2 mM) at 37°C for 2 hours. Samples were measured with a microplate reader at an absorbance of 405 nm.

### 2,5-HD treatment of VSC4.1 cells

2,5-HD treatment of VSC4.1 cells was performed as described in Liu et al. [37] with a little modification. To determine the effect of 2,5-HD on cellular apoptosis and whether NGF could protect cells from 2,5-HD toxicity via PI3K/Akt pathway, we pretreated VSC4.1 cells with DMEM containing 2% FBS for 24 hours. The cells were then treated with 10 mM 2,5-HD only, or 10 mM 2,5-HD+50 μg/L NGF, or 10 mM 2,5-HD+50 μg/L NGF+25 μM LY294002. Apoptosis of cells was detected by TUNEL, the expressions of Akt, p-Akt, Bad and p-Bad were detected by Western blotting assays.

### Statistical analysis

All results were expressed as mean ± S.D., and the statistical analysis was performed with one-way analysis of variance (ANOVA), followed by LSD or Dunnett’s multiple comparison test, which was performed using SPSS 13.0 statistical software. The differences were significant at *p < 0*.*05*. The p-values less than 0.05 were considered to be significant.

## Results

### Rats exposed to 2,5-HD exhibited progressive gait abnormalities

At the beginning, all rats exhibited a normal, unaffected gait. In the second week, the 400 mg/kg 2,5-HD-treated rats showed slight gait abnormality, i.e., unsteady gait, tip-toe walking and slight hindlimb weakness, and received mean gait score about 1.71 (Table 2). After exposure for four weeks, the 400 mg/kg 2,5-HD-intoxicated rats gradually exhibited moderate hind limb weakness, accompanied with foot splay, moderate limbspread during ambulation. On the end of exposure, the rats dosed with 400 mg/kg 2,5-HD showed severely abnormal gait (foot splay, dragging hindlimbs and inability to rear) with a score of 3.75. The 200 mg/kg 2,5-HD-treated rats produced similar but less severe neurotoxicological signs (score: 2.80). The 100 mg/kg 2,5-HD-treated rats exhibited tip-toe walking and slight ataxia and received mean gait score about 1.84. No clinical signs were observed in the control rats. The toxic effect of 2,5-HD on the rats is dose-dependent.

**Table 2:** The changes of gait score during 2,5-HD intoxication.

Weeks	Control	100 mg/kg	200 mg/kg	400 mg/kg
**0**	1.00±0.00	1.00±0.00	1.00±0.00	1.00±0.00
**1**	1.00±0.00	1.00±0.00	1.00±0.00	1.00±0.00
**2**	1.00±0.00	1.00±0.00	1.33±0.48^a^^b^	1.71±0.47^a^^b^^c^
**3**	1.00±0.00	1.51±0.53	1.82±0.42^a^	2.95±0.66^a^^b^^c^
**4**	1.00±0.00	1.63±0.52	2.45±0.52 ^a^^b^	3.12±0.60 ^a^^b^^c^
**5**	1.00±0.00	1.84±0.42^a^	2.80±0.42 ^a^^b^	3.75±0.24 ^a^^b^^c^

^a^ *p < 0*.*05*, compared with control group

^b^ *p < 0*.*05*, compared with 100 mg/kg group

^c^ *p < 0*.*05*, compared with 200 mg/kg group.

### Rats exposed to 2,5-HD exhibited slower motor neural response

The average distal latency and motor nerve conduction velocity in tails of rats in these groups is shown in Table 3 and Table 4. The DL of the rats in the control group had no changes throughout the experiment, whereas the DL of the rats in the 2,5-HD-treated groups gradually increased as the exposure was continued. In five weeks after 2,5-HD administration, the average DL of the rats exposed to 100, 200 and 400 mg/kg 2,5-HD was 4.50, 5.07 and 6.81 respectively, and significantly longer than that (3.77 ms) in the control group (*p < 0*.*05*). The average MCVs in the control group had no changes throughout the experiment, while the average MCVs in the 2,5-HD-treated groups gradually decreased as the exposure was continued. In five weeks after 2,5-HD administration, the average MCVs of the rats exposed to 100, 200 and 400 mg/kg 2,5-HD was 13.28, 10.74 and 8.56 m/s respectively, and significantly slower than that (15.41) in the control group (*p < 0*.*05*).

**Table 3:** The changes of distal latency (DL) in the tail of rats exposed to HD.

	Control	100 mg/kg HD	200 mg/kg HD	400 mg/kg HD
0	3.78±0.21	3.85±0.17	3.84±0.24	3.86±0.18
1	3.73±0.14	3.81±0.15	3.88±0.13	4.48±0.22^a^^b^^c^
2	3.76±0.16	3.85±0.23	4.00±0.14	4.55±0.17^a^^b^^c^
3	3.80±0.22	3.86±0.15	4.50±0.27^a^^b^	5.16±0.21^a^^b^^c^
4	3.75±0.17	4.06±0.21	4.67±0.32^a^^b^	5.66±0.19^a^^b^^c^
5	3.77±0.21	4.50±0.14^a^	5.07±0.21^a^^b^	6.81±0.24^a^^b^^c^

^a^ *p < 0*.*05*, compared with the control group

^b^ *p < 0*.*05*, compared with the 100 mg/kg group

^c^ *p < 0*.*05*, compared with the 200 mg/kg group.

**Table 4:** The changes of motor nerve conduction velocity (MCV) in the tail of rats exposed to HD.

	Control	100 mg/kg HD	200 mg/kg HD	400 mg/kg HD
0	15.70±0.54	15.63±0.38	15.52±0.66	15.32±0.42
1	15.66±0.84	15.10±0.57	14.92±0.61	14.48±0.59^a^
2	15.58±0.77	14.85±0.56	13.85±0.59	12.11±0.47^a^^b^^c^
3	15.61±0.52	14.75±0.58	12.84±0.57^a^^b^	11.35±0.60^a^^b^^c^
4	15.52±0.56	14.04±0.48^a^	12.43±0.64^a^^b^	10.92±0.57^a^^b^^c^
5	15.41±0.52	13.28±0.42^a^	10.74±0.52^a^^b^	8.56±0.41^a^^b^^c^

^a^ *p < 0*.*05*, compared with the control group

^b^ *p < 0*.*05*, compared with the 100 mg/kg group

^c^ *p < 0*.*05*, compared with the 200 mg/kg group.

### 2,5-HD induced apoptosis of neuron in the spinal cord of rats

**Fig 1:**
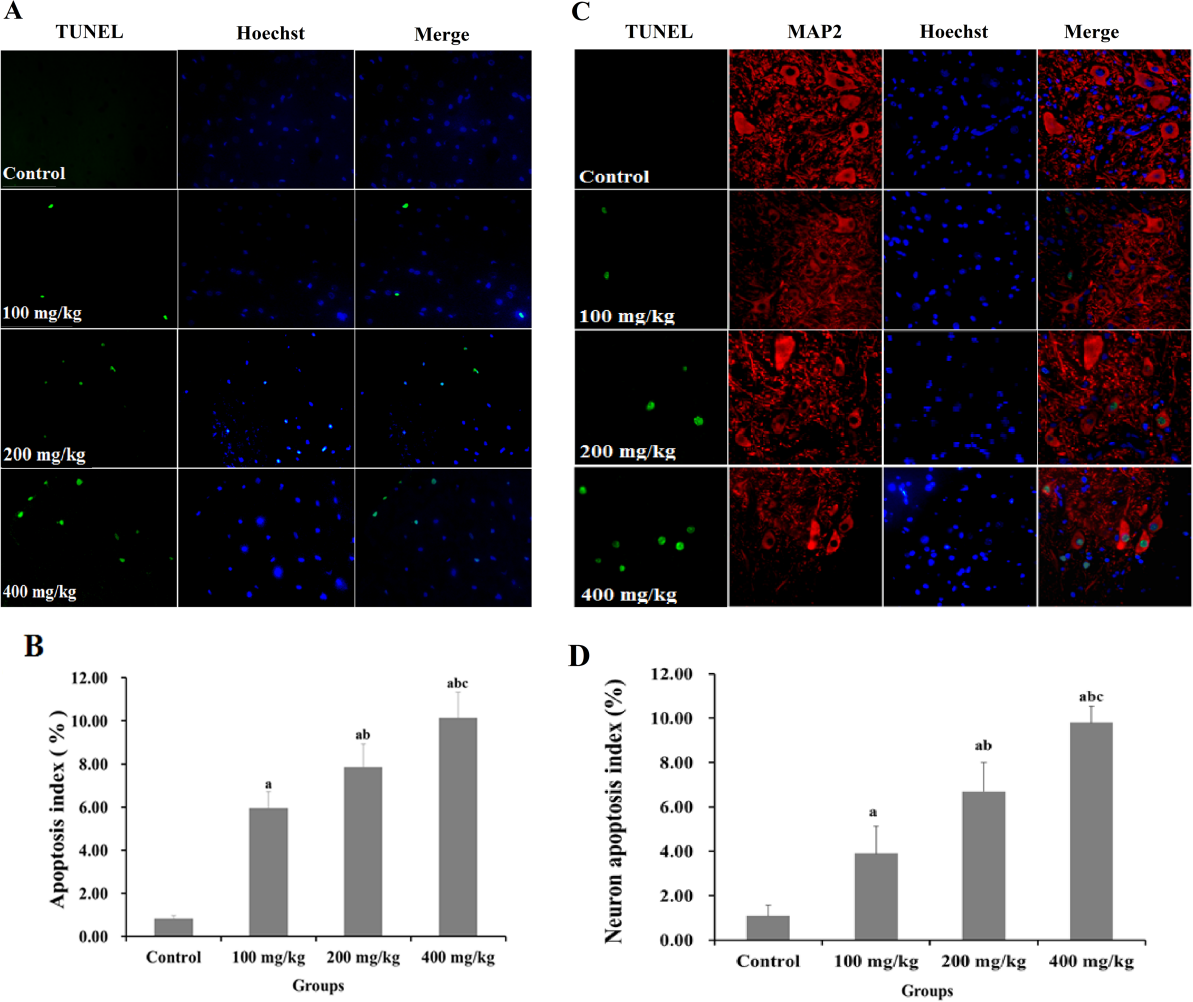
TUNEL analysis of 2,5-HD-induced cell apoptosis in the spinal cord of rats. Rats were exposed to 0, 100, 200, and 400 mg/kg 2,5-HD for 5 weeks. (**A**) Representative images of TUNEL stained cells are shown in the control group and the experimental groups. Green fluorescence represents the apoptosis cells, blue fluorescence represents the nuclei of the cells. (**B**) The apoptosis index of cells in control and the experimental groups. (**C**) Representative images of TUNEL and MAP2 stained cells are shown in the control group and the experimental groups. Red fluorescence represents the neuron marker MAP2. (**D**) The apoptosis index of neurons in control and the experimental groups. Data were shown as the mean ± SD, ^a^ *p < 0*.*05*, compared with control group; ^b^ *p < 0*.*05*, compared with 100 mg/kg group; ^c^ *p < 0*.*05*, compared with 200 mg/kg group.

Apoptotic cells in spinal cord were highlighted as TUNEL positive cells (shown in Fig 1A). They were found in the spinal cord of rats exposed to 2,5-HD, while almost absent in the control group. The apoptosis index in spinal cord of lumbar vertebra was 5.9%, 7.8% and 10.1% respectively in rats exposed to 100, 200 and 400 mg/kg 2,5-HD, significantly higher than 0.8% in the control group (*p < 0*.*05*) (Fig 1B).

We further examined apoptosis of neurons in the spinal cord by immunofluorescence dual staining (shown in Fig 1C). Neurons in the tissue slice were labeled by MAP2 antibody and shown as red. Apoptotic neurons in spinal cord were highlighted as TUNEL^+^/MAP2^+^ cells. The apoptosis index of neurons in spinal cord of lumbar vertebra was 4.1%, 6.7% and 9.8% respectively in rats exposed to 100, 200 and 400 mg/kg 2,5-HD, significantly higher than 1.1% in the control group (*p < 0*.*05*) (Fig 1D).

### 2,5-HD repressed NGF expression in the spinal cord of rats

**Fig 2:**
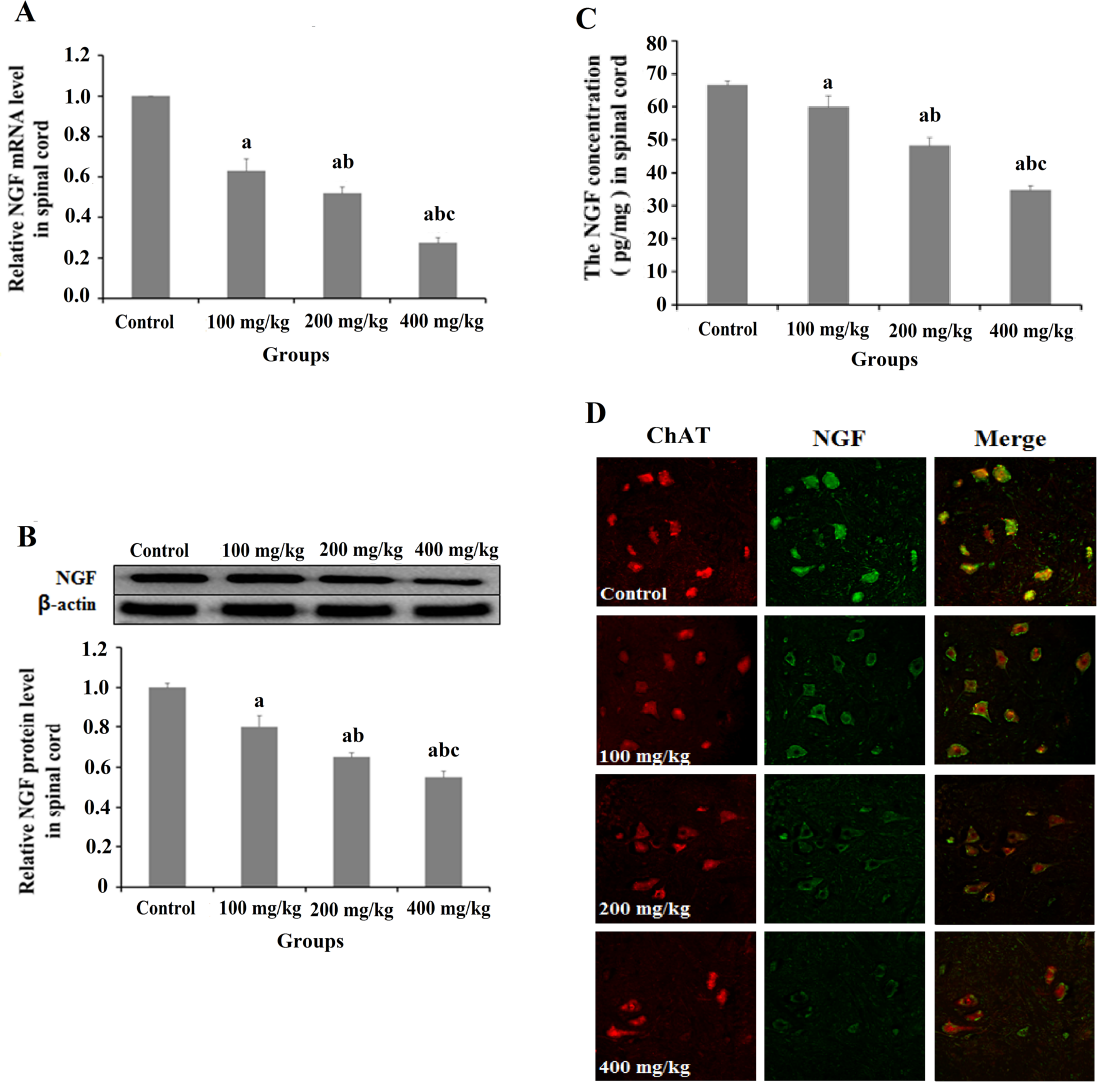
Biochemical analysis of NGF expression in the spinal cord of 2,5-HD-treated rats. Rats were exposed to 0, 100, 200, and 400 mg/kg 2,5-HD for 5 weeks. (**A**) NGF mRNA expression in the spinal cord. (**B**) NGF protein expression in the spinal cord. (**C**) The mean protein levels of NGF in the spinal cord. (**D**) Dual fluorescent staining of ChAT and NGF in the spinal cord. Red fluorescence indicates the motor neurons marker ChAT, green fluorescence represents the NGF expression. Data were shown as the mean ± SD, ^a^ *p < 0*.*05*, compared with control group; ^b^ *p < 0*.*05*, compared with 100 mg/kg group; ^c^ *p < 0*.*05*, compared with 200 mg/kg group.

The level of NGF mRNA in the spinal cord of lumbar vertebra was examined by RT-qPCR (shown in Fig 2A). The mRNA level of NGF in spinal cord was significantly lower in groups exposed to 2,5-HD than that in the control group (*p < 0*.*05*). NGF protein expression in the spinal cord was analyzed using Western blot (shown in Fig 2B). NGF protein levels in the spinal cord were significantly lower in the three experimental groups than that in the control group (*p < 0*.*05*). The NGF protein levels were also determined by commercial ELISA kit (shown in Fig 2C). The mean protein levels of NGF in the spinal cord exposed to 100, 200 and 400 mg/kg 2,5-HD were 59.9, 48.2 and 34.6 pg/mg, respectively, and significantly lower than that in the control group (66.5 pg/mg) (*p < 0*.*05*). The NGF expression in the motor neurons of the spinal cord was visualized by dual fluorescence staining of the slices with ChAT-specific (shown as red) and NGF-specific (shown as green) antibodies (Fig 2D). It was shown that NGF level in motor neurons were significantly lower in 2,5-HD treated groups than that in the control group.

### 2,5-HD repressed Akt and p-Akt, Bad and p-Bad protein expression in the spinal cord of rats

**Fig 3:**
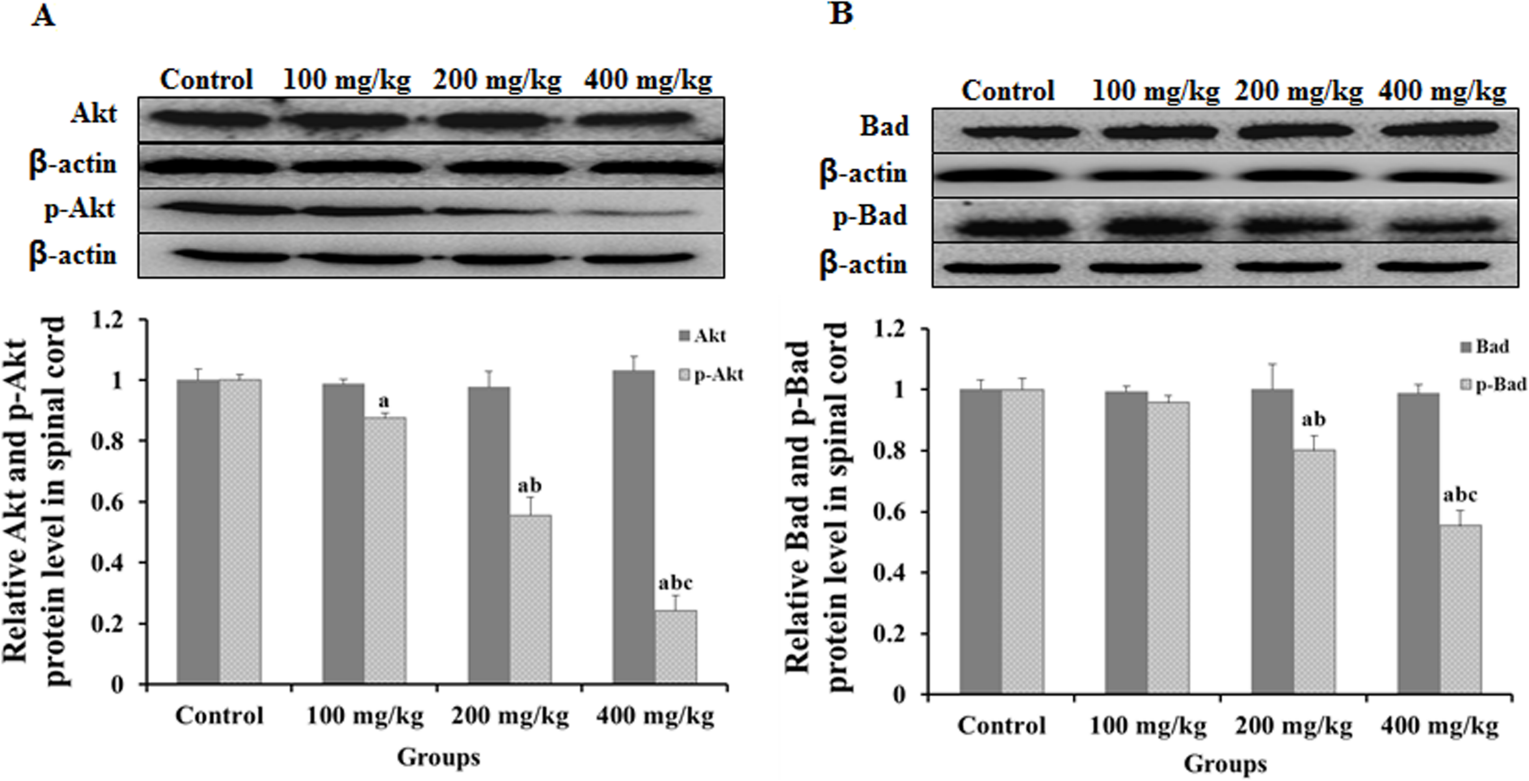
Western blot analysis of Akt and Bad expression in spinal cord of 2,5-HD-treated rats. The expressions of Akt and p-Akt, Bad and p-Bad protein were analyzed by Western blot. (**A**) The expression of Akt and p-Akt in the spinal cord. (**B**) The expression of Bad and p-Bad in the spinal cord. Data obtained from three separate analyses are expressed as mean ± SD, ^a^ *p < 0*.*05*, compared with the control group; ^b^ *p < 0*.*05*, compared with the 100 mg/kg group; ^c^ *p < 0*.*05*, compared with the 200 mg/kg group.

The expression and phosphorylation level of Akt and Bad were examined by Western blot assay using specific antibodies (Fig 3). The expression levels of Akt and Bad in the spinal cord of rats showed no significant changes in experimental groups compared with controls (*p >* 0.05). However, the amount of p-Akt and p-Bad in spinal cord was significantly lower in rats exposed to 2,5-HD than that in the control group (*p < 0*.*05*) and decreased in a dose-dependent manner.

### Influence of 2,5-HD over the key members of PI3K/Akt pathway in the spinal cord of rats

**Fig 4:**
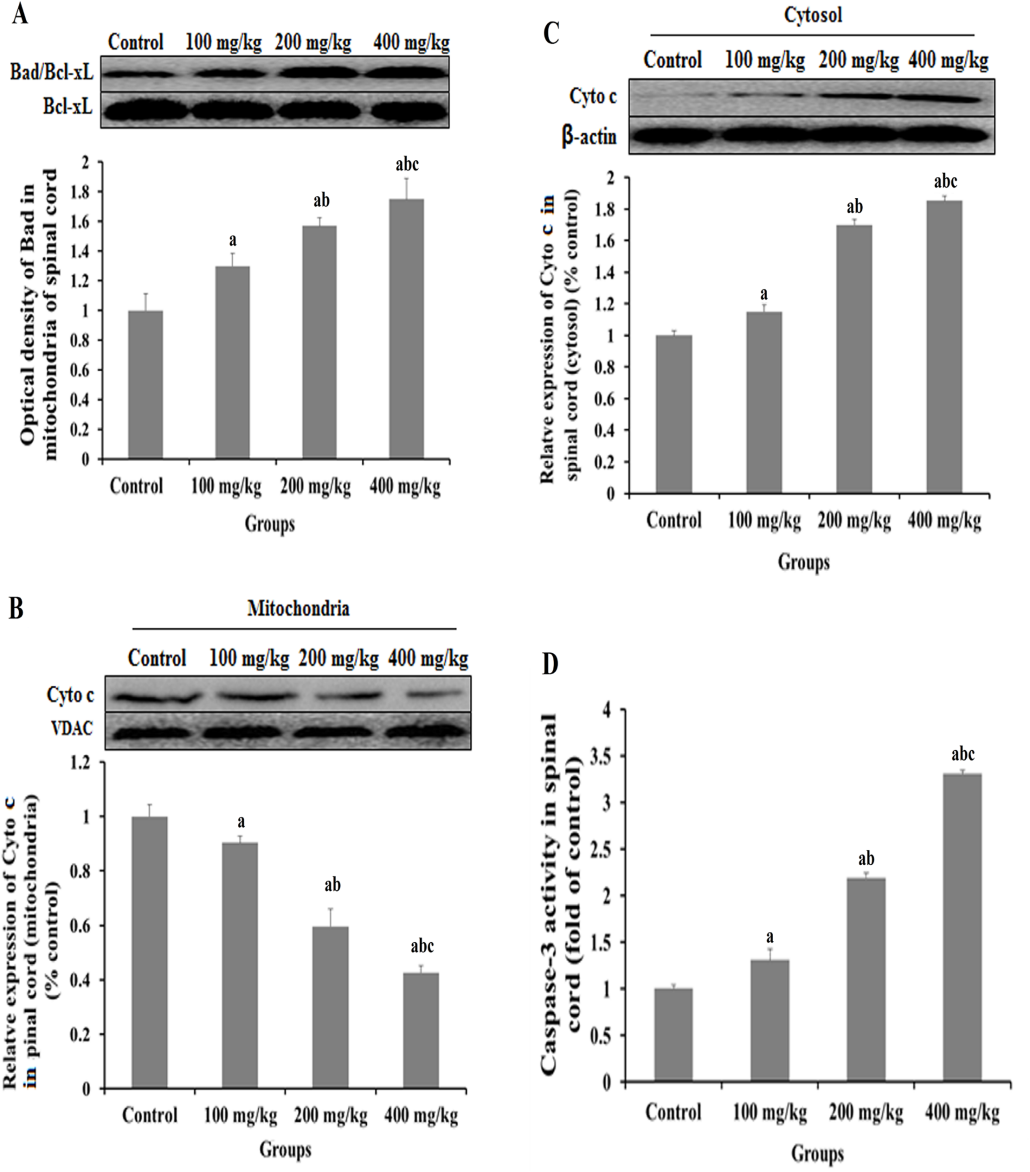
Biochemical analysis of key members of PI3K/Akt pathway in the spinal cord of 2,5-HD-treated rats. Rats were exposed to 0, 100, 200, and 400 mg/kg 2,5-HD for 5 weeks. (**A**) Coimmunoprecipitation of Bad/Bcl-xL in the mitochondrial fraction of rat spinal cord. The expressions of Cyto c (cytochrome c) protein in mitochondria (**B**) and cytosol **(C)** of the spinal cord were analyzed by Western blot. (**D**) Caspase-3 activity in spinal cord. Data obtained from three separate analyses are expressed as mean ± SD, ^a^ *p < 0*.*05*, compared with the control group; ^b^ *p < 0*.*05*, compared with the 100 mg/kg group; ^c^ *p < 0*.*05*, compared with the 200 mg/kg group.

Coimmunoprecipitation of Bad with anti-apoptotic protein Bcl-xL in the mitochondrial fraction of spinal cord was performed to examine their interaction (Fig 4A). The amount of Bcl-xL in the lysate before immunoprecipitation was determined by subjecting aliquots of the lysates to Western blot analysis as internal control. The amount of Bad that coimmunoprecipitated with the Bcl-xL proteins (IP: Bcl-xL) was determined by Western blot analysis with anti-Bad mouse monoclonal antibody (WB: Bad). Bad/Bcl-xL dimerization was significantly increased in the mitochondrial fraction of experimental groups than that in controls.

The distribution of cytochrome c protein was evaluated by Western blot in the spinal cord was performed to examine their interaction tissue of rats treated with 2,5-HD. The intensity of cytochrome c band in the mitochondrion fraction was significantly lower in spinal cord was performed to examine their interaction treated with 2,5-HD than that in control group (*p < 0*.*05*) and decreased in a dose-dependent manner (Fig 4B). On the other hand, the amount of cytochrome c protein in cytosol was significantly higher in the spinal cord of treated with 2,5-HD than that in the control group (*p < 0*.*05*) (Fig 4C).

Activity of caspase-3 in the spinal cord was analyzed (shown in Fig 4D). The activity of caspase-3 in the spinal cord was significantly higher in the groups exposed to 2,5-HD than that in the control group (*p < 0*.*05*) and increased in a dose-dependent manner. Especially, the caspase-3 activity was the highest in group exposed to 400 mg/kg 2,5-HD among the four groups.

### 2,5-HD repressed NGF expression and Bad and Akt phosphorylation in VSC 4.1 cell line

**Fig 5:**
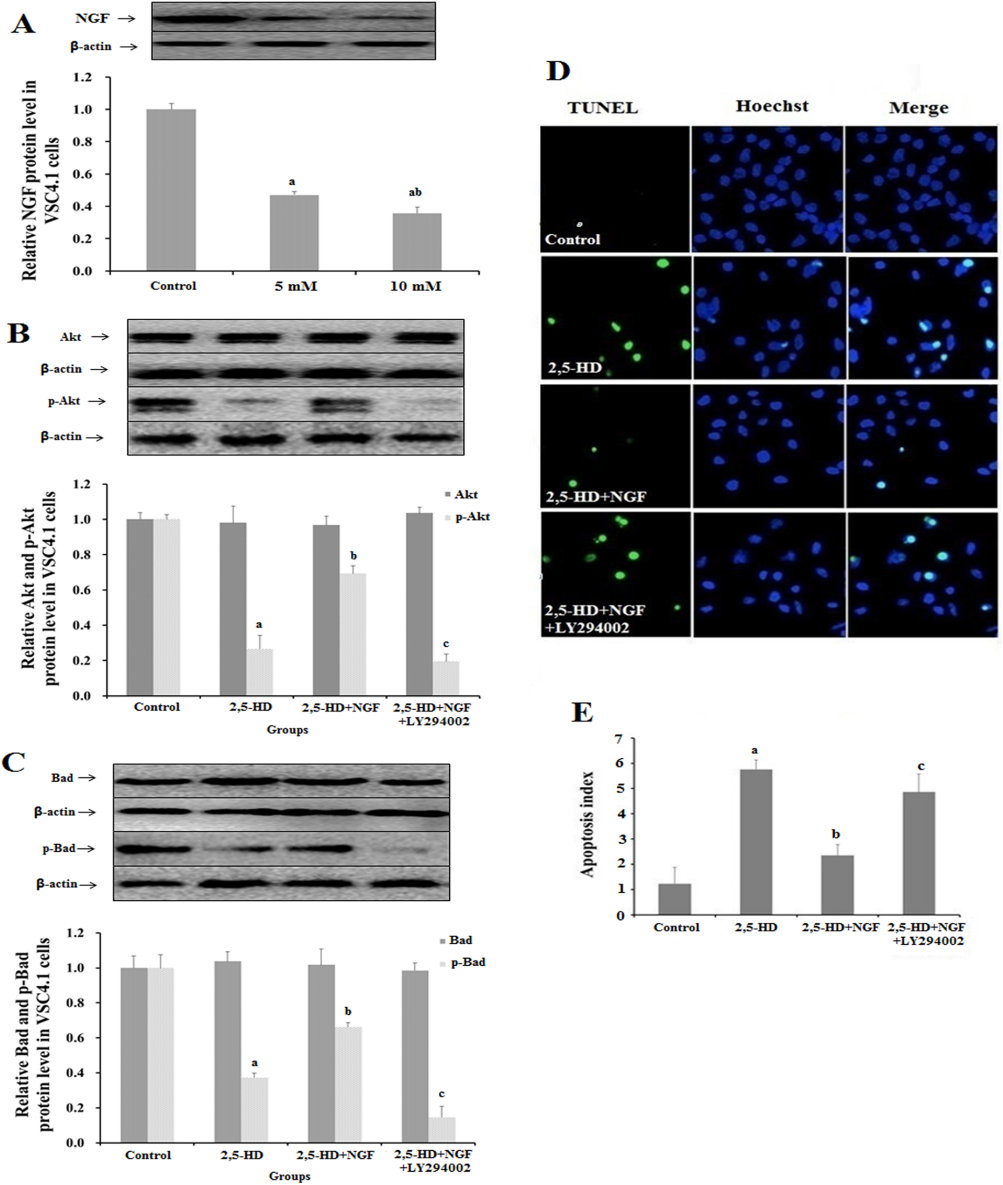
Biochemical analysis of NGF, Bad and Akt expression in VSC4.1 cell line treated with 2,5-HD. VSC4.1 cells were treated with 0, 5, 10 mM 2,5-HD for 24 h. (**A**) The expression of NGF protein in VSC4.1 cells was analyzed by Western blot. The cells were treated with 10 mM 2,5-HD, 10 mM 2,5-HD+50 μg/L NGF and 10 mM 2,5-HD+50 μg/L NGF+25 μM LY294002 for 24h. (**B**) The expression of Akt and p-Akt in VSC4.1. (**C**) The expression of Bad and p-Bad in VSC4.1. (**D**) Apoptosis in VSC4.1 cells was observed by TUNEL assay. Green color represents TUNEL-positive cells as apoptosis. Blue color represents cell nuclei counterstained with Hoechst 33342. (**E**) Bar graph indicating the percentage of apoptotic cells in each group. Data obtained from three separate analyses are expressed as mean ± SD, ^a^ *p < 0*.*05*, compared with the control group; ^b^ *p < 0*.*05*, compared with the 100 mg/kg group; ^c^ *p < 0*.*05*, compared with the 200 mg/kg group.

The NGF expression in 2,5-HD treated VSC4.1 cells was analyzed by Western Blot (Fig 5A). It was shown that in the 5, 10 mM 2,5-HD treated group, NGF expression remarkedly decreased by 48.16% and 36.57%, respectively compared with the control group, consistent with the experimental results done in the rats aforementioned.

The levels of Akt, p-Akt (Fig 5B), Bad and p-Bad (Fig 5C) proteins in ventral spinal cord 4.1 (VSC4.1) exposed to 2,5-HD were analyzed by Western blot. The p-Akt and p-Bad level decreased by 26.7% and 37.3% respectively in the 2,5-HD group compared with the control group (*p < 0*.*05*). NGF treatment of cells exposed to 2,5-HD resulted in a significant recovery of p-Akt and p-Bad to 69.3% and 66.2% respectively compared with 2,5-HD group (*p < 0*.*05*). This phenomenon could be reversed by preincubating of VSC4.1 with LY294002, which blocked PI3K/Akt pathway via inhibiting PI3K kinase. Both p-Akt and p-Bad levels dropped in this treatment group, the expression decreased by 19.5% and 14.6% compared with control group. Taken together, NGF may protect VSC4.1 cell from 2,5-HD-induced apoptosis via PI3K/Akt pathway.

To test whether NGF is involved in 2,5-HD-induced apoptosis in neurons in vitro, VSC4.1 after 2,5-HD treatment was analyzed by double-labeling experiments combining TUNEL and Hoechst 33342 staining (Fig 5D). The apoptosis index of VSC4.1 was shown in Fig 5E. Compared with the control, cells exposed to 2,5-HD showed a significant increase in apoptotic percentage (5.76%), while in the presence of NGF, less VSC4.1 cells underwent apoptosis after exposure to 2,5-HD (2.35%), suggesting that NGF protected neuron cells from 2,5-HD-induced apoptosis. To test whether NGF exerted the influence via PI3K/Akt pathway, a PI3K kinase (the upstream member of Akt) inhibitor LY294002 was used to block the PI3K/Akt pathway. It was found that NGF could not recover VSC4.1 cell from HD-induced apoptosis (apoptosis percentage rebound to 4.86%), suggesting that NGF inhibited cell death via PI3K/Akt pathway.

## Discussion

In the present study, to construct an animal model for neuropathy induced by 2,5-HD, based on references published [38], we administered 100, 200, and 400 mg/kg 2,5-HD by intraperitoneal injection to SD rats for in vivo study. The rats dosed with 400 mg/kg 2,5-HD showed foot splay, dragging hindlimbs and inability to rear. The 200 mg/kg 2,5-HD-treated rats produced similar but less severe neurotoxicological signs. The 100 mg/kg 2,5-HD-treated rats exhibited tip-toe walking and slight ataxia. Moreover, our electrophysiological results showed the average DL was significantly longer and the average MCV was significantly slower in tails of rats exposed to 2,5-HD than that in the control group. However, in the control group no clinical signs were observed and no abnormal changes of the average DL or MCV were found throughout the experiment either. These results indicate that 2,5-HD exposure have resulted in evident damage of nerve tissue and neurological deficits in the rats, consisting well with the results of other groups [39].

Apoptosis, or programmed cell death, is a highly regulated process and plays an important role in the homeostasis of nervous system. The disruption of this process can lead to abnormal increase of neuron apoptosis, contributing to the pathophysiology of various nervous system disorders [40]. Some studies showed that 2,5-HD induces apoptosis in vitro, but no observation in vivo has been reported. In the present study, apoptosis in nerve tissue was found in the rats exposed to 2,5-HD by TUNEL assays. The results showed that TUNEL-positive cells in spinal cord were significantly higher in the groups exposed to 2,5-HD than that in the control group and the apoptotic index were increased in a dose-dependent manner. Colocalization of MAP2 and TUNEL confirmed that apoptotic cells were mainly neurons. Taken together, 2,5-HD induced neuron apoptosis in the spinal cord of rats, which may be one the reasons for neurological and behavioral deficits in the experimental groups.

NGF is an important neutrotrophin for neural survival. In vitro studies showed that NGF deprivation activated the mitochondrial pathway of apoptosis in sympathetic neurons cell lines [20]. On the other hand, Shimoke et al. [19] reported that NGF administration prevented thapsigargin-induced apoptosis in PC12 cells. To examine whether 2,5-HD had any effect over NGF in nerve tissue of rats, the level of NGF in the spinal cord was investigated in the rats exposed to 2,5-HD. It was shown that both mRNA and protein levels of NGF in the spinal cord of rats were significantly lower in groups exposed to 2,5-HD than those in the control group. Moreover, the content of NGF in the spinal cord was determined by ELISA kit and found to be significantly decreased in the rats exposed to 2,5-HD, confirming that 2,5-HD downregulated expression of NGF in the spinal cord of rats.

The PI3K/Akt pathway has been reported as the downstream NGF mediated cell survival [41] via phosphorylating Bad [42]. Akt is a principal mediator in this pathway. Research has demonstrated that phosphorylation of Akt inhibits cell apoptosis [43]. It was also showed that inhibiting phosphorylation of Akt in the neonatal rat hippocampus induced neuroapoptosis. In the present study, the expression levels of Akt in spinal cord of rats showed no significant changes in experimental groups compared with the controls. However, the phosphorylation level of p-Akt in the spinal cord was significantly lower in rats exposed to 2,5-HD than that in the control group with a dose-dependent manner. Our results indicated that 2,5-HD inhibited Akt phosphorylation in the spinal cord of rats, suggesting that inhibiting PI3K/Akt signaling pathway may be a mechanism of 2,5-HD-induced apoptosis in the nervous tissue of rats.

Bad is a key member of Bcl-2 family and can be phosphorylated by Akt [21]. Unphosphylated Bad binds with anti-apoptotic proteins Bcl-2 and Bcl-xL [44–45], and suppresses cytochrome c release from mitochondria and consequently inhibiting apoptosis [45–46]. In the present study, in order to study downstream of the Akt signaling pathway, the expression levels of Bad and p-Bad in the spinal cord of rats were investigated. There was no significant change of Bad expression in the spinal cord of rats between experimental groups and the control group, whereas the phosphorylation level of Bad in spinal cord were significantly lower in the rats exposed to 2,5-HD. These results indicated that 2,5-HD treatment repressed Bad phosphorylation in the spinal cord of rats. To check the dimerization of Bad with Bcl-xL, coimmunoprecipitation approach was performed. It was found that in the nerve tissues of rats intoxicated with 2,5-HD, more unphosphorylated Bad molecules were bound with Bcl-xL. Furthermore, when mitochondrion fraction was carefully separated from cytosol, we found cytochrome c were more in the cytosol, and less in the mitochondria of the nerve cells of rats treated with 2,5-HD as the concentration of the toxin increased. Agreeing well with the results above, activity of caspase-3, the direct trigger of apoptosis, was found to be much higher in the nerve tissue of the rats exposed to 2,5-HD than that in the control group. Taken together, the changes of the major downstream members of PI3K/Akt pathway implied that 2,5-HD-induced apoptosis in nervous tissue of rats was via PI3K/Akt pathway and the subsequent mitochondrial apoptotic events, in which NGF might be involved.

To verify the experimental results in vivo, we performed in vitro study using VSC4.1 as the model, and used 5 and 10 mM 2,5-HD to treat the cell based on the references published [37,47–48]. After 2,5-HD treatment NGF expression was decreased remarkably in VSC4.1, consistent with the experimental results in the spinal cord of rats exposed to 2,5-HD.

Zhang et al. [49] showed that VSC4.1 cells were treated with 2,5-HD at doses of 10 mM and 20 mM for 12 hours, when compared with the control group, the expressions of NGF of VSC4.1 cells decreased (33.5%, 34.0%), consistent with our results. It was found that 2,5-HD treatment also reduced phosphorylation of Akt and Bad in VSC4.1 cells, and consequently induced cell apoptosis. Addition of NGF could recover p-Akt and p-Bad levels and also increased cell viability in 2,5-HD treated VSC4.1 cells, consistent with the experimental results in the spinal cord of rats exposed to 2,5-HD. LY294002 is a PI3K inhibitor that was used to block PI3K/Akt pathway, because PI3K is upstream of Akt on this pathway. When 2,5-HD-treated VSC4.1 was incubated with NGF together with LY294002, NGF could not exert its function via PI3K/Akt pathway. We found that NGF had no protective function over VSC4.1. This result confirmed that NGF inhibited cell apoptosis induced by 2,5-HD via PI3K/Akt pathway.

In conclusion, the present study showed that 2,5-HD exposure repressed NGF expression and induced apoptosis in the spinal cord of rats via deactivating PI3K/Akt signaling pathway. The similar effect was also observed in vitro. It is thus suggested that inhibiting PI3K/Akt signaling pathway may be a mechanism of 2,5-HD-induced apoptosis in nervous tissue of rats, which may be associated with NGF repression by 2,5-HD.
